# Adapting research ethics for global health crises: a systematic analysis of COVID-19 pandemic research guidelines and reflections on their post-pandemic implications

**DOI:** 10.1186/s12910-025-01371-6

**Published:** 2026-01-10

**Authors:** Vilma Lukaševičienė, Eugenijus Gefenas

**Affiliations:** Faculty of Medicine, Institute of Health Sciences, Centre for Health Ethics, Law and History, Vilnius University, Vilnius, Lithuania

**Keywords:** Research ethics, Research ethics committee, COVID-19 pandemic.

## Abstract

**Background:**

the COVID-19 pandemic posed unprecedented challenges to global research ethics governance. In response to the urgent need for rapid scientific data, ethics review processes were accelerated, informed consent procedures adapted, and new research methods introduced. These changes were guided by the proliferation of research ethics guidelines issued by national, regional and international bodies. However, there has been limited systematic analysis of how these guidelines addressed key ethical challenges during the pandemic and shaped future ethical frameworks. This study aims to map the global network of pandemic related research guidelines, to analyze how they addressed the main pandemic specific ethical challenges.

**Methods:**

We conducted a systematic search of published research ethics and regulatory guidelines developed in response to the COVID-19 pandemic between 2020 and 2022. We applied a mixed methods approach: citation network analysis was used to visualize interconnections between guidelines and identify influential sources, and a thematic analysis was used to examine how guidelines addressed specific ethical issues.

**Results:**

the citation network analysis revealed a globally interconnected ethical framework with the WHO outbreak guidelines (2016) and CIOMS guidelines (2016) emerging as central references. Thematic analysis identified eight recurring ethical themes: research prioritization, research ethics oversight, inclusion of vulnerable populations, balancing risks and benefits, modifications of informed consent, data sharing and transparency, collaboration and preparedness, and public engagement. Guidelines encouraged procedural flexibilities (e.g., expedited reviews, e-consent) but also emphasized the need to maintain ethical rigor and scientific integrity. Tensions between speed and scrutiny, centralization and local context, public health goals and individual protection were recurring concerns. Integration of public health ethics, research integrity, and human rights frameworks emerged as a defining feature of most guidelines.

**Conclusions:**

COVID-19 catalyzed a shift toward more integrated and adaptive research ethics frameworks that combine individual protection and collective public health goals. These shifts are being reflected in recent updates of major ethics instruments such as the Declaration of Helsinki and new policy initiatives such as the European Health Data Space Regulation. However, gaps remain in harmonization, implementation, and preparedness planning. Continuous efforts are needed to harmonize ethics guidance, support research ethics committees in crisis contexts, and integrate preparedness planning into global research ethics governance.

**Supplementary Information:**

The online version contains supplementary material available at 10.1186/s12910-025-01371-6.

## Background

Six years have passed since the COVID-19 pandemic began and created an unprecedented global health crisis, challenging medical systems, research infrastructures, and research ethics frameworks worldwide. Although there has been a firm theoretical foundation for research ethics in crisis situations guided by established frameworks, including the Declaration of Helsinki, Good Clinical Practice (GCP) guidelines, and WHO ethical research standards [1], the pandemic revealed insufficiencies in ethical governance [2] and necessitated its adaptations [3].

Reacting to this need for research, global regulatory bodies, including the Food and Drug Administration (FDA), the European Medicines Agency (EMA) and the World Health Organization (WHO), implemented regulatory flexibility to fast-track research and improve review efficiency [4]. Research Ethics Committees (hereafter - RECs) were also forced to accelerate review processes to facilitate rapid research response [5], but such adaptations led to other ethical challenges such as reduced scrutiny in research protocols [6], increased workload for RECs [7], ethical inconsistencies across jurisdictions [8], leading to concerns about review quality. Concerns arose regarding reduced scientific rigor, the potential for increased data fabrication and rushed publications leading to later research retractions [9] and the role of preprints and unreviewed studies in misinformation dissemination [10].

The COVID-19 pandemic also reshaped research priorities, often sidelining studies unrelated to the virus. While prioritization was necessary, it raised ethical concerns regarding equity in research funding allocation [11], exploitation of vulnerable populations [12], the long-term impact of COVID-centric research on other critical fields such as cancer [13] or mental health research [14].

The COVID-19 pandemic necessitated alterations to informed consent processes, particularly in intensive care settings and for vulnerable populations [15]. Key adaptations included e-consent and verbal consent procedures to reduce physical contact, proxy consent or deferred consent for emergency interventions. While these modifications ensured research continuity, questions remain about participant vulnerability and potential coercion [16, 17].

The ethical guidelines developed during the pandemic had to address all these challenges and reconcile two seemingly opposing imperatives: the need for speed in generating and disseminating knowledge, and the duty to uphold core research ethics principles. While some scholars argued for ethical “exceptionalism”, suggesting that the unique circumstances of the pandemic warranted a relaxation of certain research ethics standards [18, 19], others contended that even in times of crisis, adherence to foundational research ethics principles is paramount and that compromising ethical safeguards would undermine public trust and scientific integrity [1, 20, 21] .

In the post-pandemic period, the lessons learned are catalyzing changes in global research ethics governance. However, to our knowledge, there has been no systematic analysis of how COVID-19 specific ethics guidelines navigated the ethical challenges of the pandemic. Also, it seems there has been little reflection on the implications of the lessons learned on the post-pandemic developments. This study addresses these gaps by examining the landscape of research ethics guidelines developed in response to COVID-19. The objectives are twofold: (1) to map the network of pandemic research ethics guidelines, exploring their interconnections and influences in shaping a cohesive global framework; and (2) to explore how these guidelines addressed key ethical challenges in pandemic research and their implications for the post-pandemic development of research ethics.

As time passes, it becomes harder to prioritize preparations for future pandemics, but scientists urge to “not let history be forgotten” [22], and call for a permanent emergency ethics review frameworks to be established to prevent ethical shortcuts in future pandemics [23]. By analyzing both the structure and content of the pandemic guidelines, we aim to provide insights that could inform more resilient and adaptable research ethics frameworks for future global health emergencies.

## Methods

The study employed a mixed-methods approach, combining network analysis and thematic analysis to comprehensively map and evaluate the ethical guidelines developed during the COVID-19 pandemic. Each method provided a distinct lens for understanding the structure and content of pandemic ethics documents and the combination of these methods helps to better understand how research ethics frameworks were adapted under the pressure of COVID-19.

### Systematic document search

**Fig. 1:**
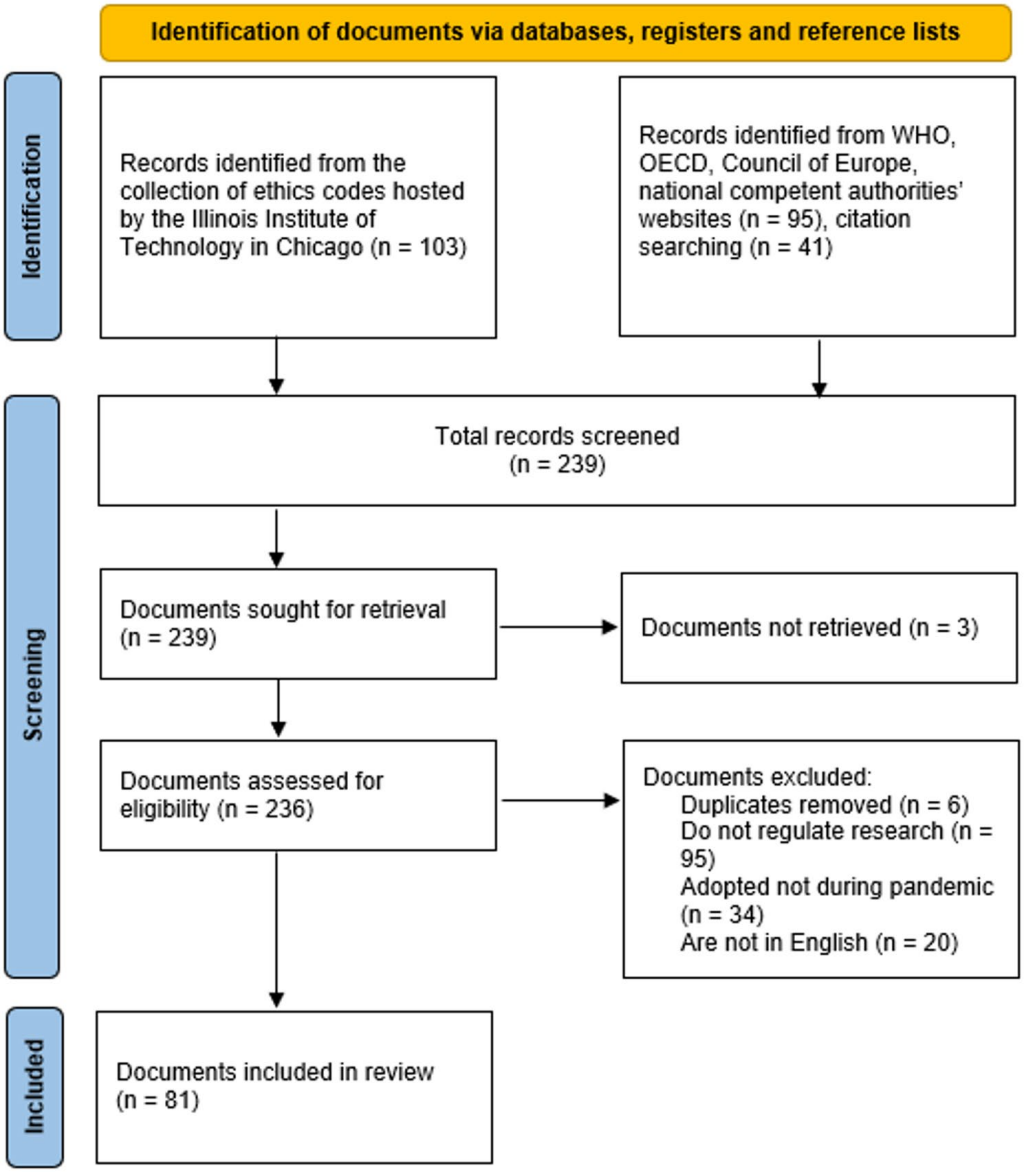
PRISMA flow diagram of the systematic literature search [24]

The article draws substantially on findings from the EU-funded project PREPARED (Pro-active Pandemic Crisis Ethics and Integrity Framework) that aims to develop an operational ethics and integrity framework, which safeguards key ethical values, supports a rapid and effective research response to crises and improves overall pandemic preparedness^1^. The search for relevant guidelines was conducted in two stages. First, a comprehensive search was conducted in the Illinois Institute of Technology library, which hosts one of the largest collections of ethics codes and guidelines in the world. This search identified 103 potentially relevant documents, focusing on guidelines issued by international, national, and professional bodies. A second search, which included examining reference lists and scanning the databases of European and global health agencies, yielded an additional 136 documents. The combined search resulted in a pool of 239 pandemic documents, which were then manually reviewed to remove duplicates and ensure that each document met the inclusion criteria. To be included, documents had to be: (a) published during the COVID-19 pandemic (2020–2022); (b) explicitly addressing the ethical conduct of research in the context of the pandemic; (c) issued by an international, regional, or national authority or professional body; and (d) available in English (Fig. 1).

This selection process resulted in 81 documents being included in the final analysis, ranging from globally applied international documents to detailed institutional ethics and regulatory guidelines.^2^

### Network analysis

To identify which guidelines were most influential and how a global research ethics framework for the pandemic emerged through cross-referencing and adoption of common provisions, we used citation network analysis (CNA) [25]. This allowed us to explore relationships between the identified guidelines, focusing on how often these documents reference each other. For each of the 81 guidelines, we extracted all citations of other guidelines or policy documents (explicit mentions in the text, footnotes or references), compiling a database of these inter-document references. We then constructed a citation network graph using Gephi software, where each guideline is a node and each citation is a directed edge. We applied the Fruchterman-Reingold and Force Atlas 2 layouts to visualize the network, which positions nodes based on their citation connections. Guidelines (nodes) frequently cited by many others gravitate to the center of the network, highlighting them as influential guidelines that shaped the pandemic research ethics discourse.

### Thematic analysis

In addition to network analysis, a thematic analysis of the selected documents was conducted to examine the specific ethical issues and recommendations emerging in the pandemic guidelines [26]. Using MAXQDA software, we coded each guideline document for key themes and ethical topics. We followed a hybrid inductive-deductive coding approach [27]: initial codes were generated inductively from reading a subset of guidelines, and a preliminary codebook of recurrent themes was created. We then applied this codebook to other documents, refining and adding codes if new sub-themes emerged. This qualitative analysis enabled us to compare how different guidelines addressed the same ethical challenges and to identify common standards or notable divergences. We identified eight general themes prevalent in COVID-19 pandemic guidelines, which will be discussed in the Results section. We draw specific attention to how pandemic-specific guidelines aligned with established pre-pandemic ethics guidelines in order to determine whether they upheld foundational principles such as balancing risks and benefits, informed consent or ethics oversight and to identify any new topics that emerged. We also examined whether and how the guidelines addressed ethical challenges heightened by COVID-19 and whether they introduced new or adaptive mechanisms tailored to crisis conditions.

## Results

### General description of the guidelines

Guidelines were adopted by both international organizations as well as national bodies. Many are dedicated ethics bodies or public health organizations, such as the WHO, UNESCO, building global frameworks, or regional collaborations (Pan American Health organization (PAHO), European Network of Research Ethics Committees (EUREC). National regulatory authorities and research oversight bodies also played a prominent role in pandemic research governance. These include drug and health product regulators (e.g. FDA, EMA, Medicines and Health products Regulatory Agency (MHRA), South African Health Products Regulatory Authority (SAHPRA), national research institutes and councils (National Institutes of Health (NIH), Uganda National Council for Science and Technology (UNCST), Kenya Medical Research Institute (KEMRI). Most of the guidelines adopted by regulatory bodies were of the national level. This suggests the regulatory guidance was decentralized (with the exception of EMA guidelines on clinical trials on medicinal products in the European Union (EU) region), while ethical guidance had a mix of local and international sources, pointing to efforts at global ethical consensus during COVID-19.

Most documents are around 15–17 pages long. However, the ethics guidelines vary from short guidance briefs (e.g. 4 pages WHO ethical standards brief) to in-depth ethical frameworks (a comprehensive up to 156 pages COVID-19 ethics handbook from Ethiopia), whereas the regulatory guidelines tend to be more uniform in size (mostly between 10 and 40 pages) and generally are focused on specific issues.

Guidelines also vary in terms of the target groups. While regulatory guidelines target primarily researchers and regulators, ethics guidelines extend beyond researchers to healthcare professionals, policymakers, and the public, reflecting wider societal ethical concerns (Supplement 1).

### Global network of pandemic research ethics guidelines

The network graph of the 81 documents, visualized using CNA, reveals an interconnected global framework of pandemic research ethics guidelines, with certain documents serving as central nodes of influence (Supplement 2). Notably, several pre-pandemic international research ethics guidelines became capstones during COVID-19. The WHO guidance on managing ethics in infectious disease outbreaks (2016) [28] and the CIOMS International Ethical Guidelines for Health-Related Research (2016) [29] were the most frequently cited sources across the COVID-19-specific documents. These influential documents not only provided ready-made ethical frameworks that could be applied to COVID-19 research, but they also shaped the development of new, pandemic-specific guidelines.

Additionally, the EMA guidance for managing clinical trials during the pandemic [30] (first issued in 2020) was one of the most often cited references for regulatory documents adopted by national competent authorities in EU countries, reflecting its practical importance for clinical trials conducted under crisis conditions in EU Member States. Other frequently cited references included the Nuffield Council on Bioethics (UK) report on research in global health emergencies (2020) [31] and the World Medical Association (WMA) Declaration of Helsinki (originally adopted in 1964 and amended multiple times, the latest pre-pandemic amended version was adopted in 2013) [32]. The Nuffield Council report, though developed just before COVID-19 (published in January 2020), provided a rich discussion of ethical issues in epidemics and was widely invoked. The presence of the WMA Declaration of Helsinki in the CNA underscores that even during a crisis, researchers and RECs are expected to anchor their actions in well-established ethical principles. Our analysis also shows that many COVID-19 research ethics guidelines explicitly drew not only on traditional research ethics norms but also principles from public health ethics, clinical ethics, and human rights frameworks. This indicates that the ethical framework for conducting research during a pandemic combined individual-focused research ethics, clinical ethics and the broader societal perspective of public health ethics.

Thus, the CNA showed a dense clustering of citations internationally, with WHO and CIOMS guidelines at the center of the network, connected to other pandemic guidelines, as many national bodies referenced these global documents when crafting their own COVID-19 research ethics policies. Regional exchanges were also evident – for instance, the EU national regulatory agencies cited EMA guidelines frequently, indicating coordination and mutual learning within the EU. The CNA highlights that despite the rapid proliferation of guidelines, they were not developed in isolation but rather formed a web of reinforcing references, showing a convergence towards a shared core of ethical standards.

It is worth mentioning, however, that the visualization tools used for network analysis (e.g., Gephi’s Force Atlas and Fruchterman–Reingold layouts) influenced the perceived proximity and prominence of documents in the network. These visual patterns were used for interpretation, but should not be confused with normative hierarchy or implementation strength of the guidelines.

### Main ethical themes in COVID-19 research guidelines

Based on the thematic analysis, we distinguished eight major themes reflected in pandemic guidelines (Supplement 3). Some themes, such as balancing risks and benefits or informed consent appeared in nearly all guidelines as foundational ethical standards and other themes, such as public engagement or preparedness for future emergencies, were less frequent and more often included in guidelines with broader strategic or public health orientations. While this might indicate how different guideline developers prioritized ethical concerns during the pandemic, our data did not reveal any normative hierarchy of these themes. Therefore, the themes are presented following the stages of the thematic analysis methodology to reflect the key requirements consistently addressed across the pandemic guidelines.

#### Research prioritization

One of the key themes that emerged from the thematic analysis was the prioritization of research during the pandemic. There were 3 major subthemes emphasized.

First, guidelines worn that resources allocated to research must not be taken away from routine health care and public health services: research should never impede emergency response efforts. This means that research should not be conducted if it can be expected to take away personnel, equipment, facilities, and other resources from those required for outbreak response. Yet guidelines also recognized that research is a “key aspect of response to public health emergencies” and there is an ethical obligation to conduct research.

Second, guidelines dealt with the prioritization of research projects. On the one hand, the pandemic-specific guidelines frequently emphasized the need to prioritize research directly related to COVID-19, that aims to understand the disease, improve prevention and to assess the safety and efficacy of any proposed diagnostic tests, treatments or vaccines. However, some other guidelines raised concerns about the exclusion of non-COVID-19 research, particularly studies addressing other critical health issues that may have been deprioritized or delayed due to the focus on the pandemic.

Third, pandemic documents stated that mechanisms should be in place to identify which studies are related to the health emergency and should be prioritized. Some guidelines provided examples of such research or comprehensive lists of research areas of key importance^3^. Others suggested criteria to identify and prioritize pandemic research, for example, the impact of the proposed research on patient and participant well-being and institutional resources, consistency with response efforts and the impact on the health system or the priorities and acceptability to the community that will host it, more generally, or specific circumstances, such as the nature of the investigational and other products, the ability to conduct appropriate safety monitoring, the potential impact on the investigational and other products supply chains, and the nature of the disease under study.

Recommended research prioritization mechanisms also included practical measures such as the establishment of research “triage” committees, composed of experts tasked with reviewing and prioritizing research proposals based on their potential public health impact, entrusting the relevant research authorities to coordinate research efforts in emergencies, including the establishment of research priorities; coordination of research projects nationally and internationally to avoid wasteful duplication and underpowered studies.

#### Research ethics oversight

The rapid pace of research during the pandemic placed significant pressure on RECs. RECs were encouraged to maintain their role as independent reviewers of research proposals, ensuring that ethical standards were upheld even in the context of a rapidly evolving public health crisis. However, many of the pandemic-specific guidelines also called for the modification of REC processes to accommodate the urgent need for ethics review of a large number of COVID-19-related research proposals.

Virtual REC meetings, expedited review processes, and the use of reduced quorums was among the measures recommended to ensure that research could proceed without unnecessary delays. However, the guidelines also emphasized that these modifications should not compromise the rigor of ethical oversight.

Guidelines also suggest new approaches, such as “deferred approval”. For example, according to the joint statement ”COVID-19: Guidance on clinical trials for institutions, HRECs, researchers and sponsors,” adopted by the members of the Clinical Trials Project Reference Group in Australia if an REC considers new proposed research to be inadvisable in the current environment, the REC may choose to indicate an in principle acceptance of the merits and design of the research, but defer its approval until circumstances permit approval and commencement of the research. A prominent feature of pandemic guidelines was the call for centralization of research governance and ethical review processes. Many countries adopted a more streamlined approach, where RECs worked in coordination with national and international bodies to expedite the approval of COVID-19-related research.

#### Inclusion of vulnerable populations

Pre-pandemic ethical frameworks, such as those developed by CIOMS and the WHO, emphasized the need to protect vulnerable populations from exploitation, but also to ensure their access to the potential benefits of research. During the pandemic, this issue was further complicated by the heightened vulnerability of certain groups, such as the elderly, individuals with underlying health conditions, and marginalized communities.

The inclusion of vulnerable populations in pandemic research was another prominent theme across the guidelines. Pandemic guidelines emphasized the importance of fair selection processes, cautioning against the routine exclusion of vulnerable populations from research unless there was a strong scientific or ethical justification. This was particularly relevant in vaccine trials, where the inclusion of high-risk populations was critical for generating data on the safety and efficacy of vaccines across diverse demographic groups. Public involvement plans, designed to engage communities in the research planning process, became a requirement in ethics guidelines, ensuring that the voices of vulnerable populations were heard and that research was responsive to their needs.

#### Balancing risks and benefits

The pandemic-specific balancing of risks and benefits was also highly emphasized during the pandemic. Although risk-benefit balancing is foundational in research ethics, the guidelines stressed the need for continuous risk-benefit assessments, given the evolving nature of the pandemic and the uncertainty surrounding new treatments and interventions. Several documents recommended adaptive trial designs, which allowed for the modification of research protocols as new evidence emerged, ensuring that risks were minimized while potential benefits were maximized.

A particular focus was placed on the safety of participants and researchers, and sponsors were encouraged to implement rigorous monitoring of ongoing trials to ensure that any emerging risks were quickly identified and addressed.

#### Modifications to informed consent processes

The pandemic necessitated significant modifications to the traditional informed consent process, particularly in cases where face-to-face interactions were not feasible due to lockdowns and other quarantine measures, or the need to minimize exposure to the virus. Many guidelines introduced flexible approaches to obtaining consent, including electronic consent (e-consent), oral consent with witnesses, and deferred consent in emergency situations. These modifications were designed to balance the need for informed consent with the practical challenges posed by the pandemic.

However, the guidelines also emphasized that any deviations from standard consent procedures should be carefully justified and documented. Despite these modifications, the core ethical requirement for voluntary and informed consent remained intact. Researchers were expected to ensure that participants were fully aware of the potential risks, benefits, and purposes of the research, even when alternative methods of obtaining consent were employed. Continuous consent, wherein participants are regularly informed about new risks and developments in the study, was recommended as a best practice to ensure that consent remains informed and voluntary throughout the research process.

#### Data sharing and transparency

Data sharing emerged as a critical ethical concern during the pandemic, as the rapid dissemination of research findings was essential for informing public health responses and accelerating the development of treatments and vaccines. The pandemic-specific guidelines promoted open-access publishing, the use of preprint servers, and the immediate sharing of research data, particularly for studies with significant public health implications.

However, this push for rapid data sharing raised concerns about data quality, intellectual property rights, and the protection of participant privacy. Several guidelines stressed the importance of ensuring that shared data was accurate and had undergone sufficient quality control before dissemination. Ethical considerations around the privacy of participants were highlighted. Researchers were encouraged to adopt transparent data-sharing agreements that included provisions for maintaining confidentiality and protecting sensitive information, in line with existing data protection regulations such as the General Data Protection Regulation (GDPR) in the European Union.

The guidelines also underscored the importance of accountability in data sharing. Researchers, sponsors, and publishers were urged to ensure that data was shared responsibly, with clear mechanisms for correcting errors or retracting inaccurate information. The emphasis on transparency and accountability was seen as crucial for maintaining public trust in the scientific process, especially in the context of widespread misinformation and skepticism about COVID-19 research.

#### Collaboration and preparedness

The pandemic guidelines emphasized the importance of international and interdisciplinary collaboration in research and frequently called for the creation of networks of experts and institutions to share knowledge, coordinate research efforts, and avoid redundant or conflicting studies. This collaborative approach was seen as essential for addressing the global nature of the pandemic.

Preparedness for future pandemics was another key theme that emerged from the analysis. Several guidelines emphasized the need for the development of contingency plans and generic research protocols that could be quickly adapted to new health emergencies. This included the creation of ethical frameworks that could guide research during future pandemics, ensuring that the lessons learned from COVID-19 would be integrated into future responses. The establishment of communication mechanisms between RECs, regulatory bodies, and public health authorities was seen as critical for ensuring that ethical review processes could be rapidly mobilized in the event of a new global health crisis.

#### Public engagement and communication

The importance of public engagement and communication emerged as a distinct feature of pandemic research guidelines. Building trust with the public through transparent and respectful communication was deemed essential for the success of research initiatives. Guidelines emphasized the need for researchers to engage with communities early in the research process.

Public engagement was critical for ensuring that research protocols were aligned with the needs and values of local communities. In addition, clear and honest communication about research findings was necessary to combat misinformation and build public confidence in scientific research, particularly in the context of vaccine development. Pandemic guidelines required researchers to disseminate results transparently, not only to the scientific community but also to the broader public, using accessible language and platforms.

Public engagement was also linked to the research ethics and integrity principle of respect for persons. Researchers were encouraged to communicate openly about the risks and benefits of participation, ensuring that participants could make informed decisions even in the face of rapidly evolving information.

## Discussion

As highlighted by the CNA and our thematic analysis, pandemic guidelines mostly relied on the traditional research ethics principles of CIOMS guidelines, the WMA Declaration of Helsinki, and the WHO guidelines on infectious diseases. However, the COVID-19 pandemic put the global research ethics framework to the test and revealed areas where these traditional guidelines needed updating, from incorporating research integrity safeguards to integrating public health ethics considerations.

While pandemic guidelines frequently drew on traditional ethical frameworks, adaptability emerged rapidly in practice, as there was a notable shift from simply referencing existing, often general, ethical standards to the rapid development of new, context-specific instruments designed to address unmet ethical needs during the pandemic. The standards of ethics were supposed not to be lowered, but the means of implementing them became more flexible and context responsive, as for example, research that took place during COVID-19, normalized the use of flexible approaches to research reviews and obtaining informed consent under constraints on in-person consent processes. The guidelines also addressed other ethical problems heightened by COVID-19, such as dealing with uncertainty about emerging risks or competing demands on healthcare resources and pressures on research prioritization and introduced new adaptive mechanisms tailored to crisis conditions, such as virtual REC meetings, reduced quorums, electronic consent, prioritization committees, or rapid data-sharing procedures. .

Numerous guidelines warned against conducting underpowered or poorly designed studies in the rush to generate data, as these would expose participants to risks without likely benefit and could lead to misinformation. Many COVID-19 studies were launched at high speed, and while this led to life-saving breakthroughs (such as vaccines in record time), it also led to challenges from poorly designed trials that wasted resources to high-profile paper retractions due to erroneous or fabricated data [33]. This illustrates that ethical requirements (like obtaining informed consent or fair participant selection) alone are insufficient if the research itself lacks integrity. A “pandemic of poor research” can erode public trust and lead to ethical failures just as surely as violations of consent or confidentiality [34]. Importantly, several themes identified in the analysis, such as data sharing, methodological quality, transparency, and collaboration across institutions, traditionally associated with research integrity appeared alongside traditional research ethics topics such as balancing risk and benefit and informed consent in the context of COVID-19. Therefore, maintaining scientific integrity was highlighted as an ethical obligation in itself. Thus, one of the key findings of our study is that research ethics and research integrity should be understood as mutually reinforcing imperatives, especially in crisis situations. Recent post-pandemic instruments also explicitly reflect this integration, as discussed later in the article.

Transparency and public engagement are very important in balancing individual and collective interests. When communities understand what research is being done and why, they are more likely to support collective efforts [35]. When researchers communicate openly (for example, about research risks), it honors the principle of respect for persons at a community level. An important legacy of COVID-19 may be stronger expectations for community consultation and participant involvement in designing emergency research, integrating the participant-centric research ethics with the community-oriented public health.

Thus, another insight is the importance of public health ethics principles in guiding research during the COVID-19 pandemic [36]. Traditional research ethics focuses largely on the researcher-participant relationship and the obligations to individual participants [37]. However, a pandemic by definition involves a threat to the entire community, requiring collective action and raising ethical considerations at the population level [38]. As our CNA results show, many pandemic-specific guidelines were informed (made references to) not only by prior research ethics codes but also by broader frameworks of public health ethics and human rights [39]. This perspective influenced COVID-19 research regulation in practical ways: it led to ethical requirements to share data internationally for the common good/public interest, to include diverse populations, so that findings would be generalizable and benefits accessible to all, and to allocate trial resources to the most pressing public health questions. It also brought attention to the duty to conduct research as an ethical imperative in a pandemic. Whereas in normal times the decision to launch a study is often seen as discretionary, in an outbreak there is an argument (grounded in public health ethics) that the medical community has an obligation to rapidly learn how to save lives. Several guidelines explicitly stated that not doing research during an outbreak can be unethical. At the same time, the urgency to help the many does not trump the rights of the few. The guidelines consistently affirmed that individual participants’ rights and well-being could not be sacrificed for rapid results. For instance, even if restrictive lockdowns were in place, researchers still needed to obtain voluntary informed consent.

In looking at how these insights have been shaping post-pandemic guidelines and regulations, several developments are worth mentioning. One of the notable tendencies is the modernization of existing guidelines. The World Medical Association’s recent revision of the Declaration of Helsinki (2024) might be seen as a direct response to contemporary challenges illuminated by COVID-19. This ”post-pandemic” update to the Declaration includes several new provisions. The Declaration explicitly refers to the issues of research integrity and states that *“scientific integrity is essential”* and that researchers must never commit or engage in research misconduct [40]. The revised Declaration of Helsinki also acknowledges the context of data-intensive research. By incorporating areas traditionally considered within the domain of research integrity, such as scientific integrity, methodological robustness or responsible data sharing directly into the ethical framework and placing them alongside established ethical principles, it signals that integrity is part of ethical evaluation rather than a parallel domain. It also adds provisions about the importance of community engagement in research planning [41], incorporates concepts of global justice and equity, and calls for inclusion of under-represented groups and access to research benefits globally. By formally expanding its scope to issues of scientific integrity and public health, the Declaration of Helsinki sets a tone for ethics in the post-COVID era.

One of the major needs during COVID-19 was for rapid data (e.g., genetic sequences of the virus, patient outcomes, or trial results) sharing across institutions and borders. However, data sharing was hampered by legal and technical barriers, including privacy regulations and a lack of interoperability between health systems. This strengthened calls for more harmonized frameworks and accelerated the ongoing initiatives on data sharing, such as the establishment of the European Health Data Space (EHDS), a regulatory framework to facilitate the secure sharing of electronic health data, including for research and public health [42]. While the EHDS Regulation entered into force in March 2025, most obligations will begin to apply four years after its entry into force, with some provisions implementable by EU Member States on a voluntary basis. It is intended to create a trusted platform where researchers can access health datasets from across EU Member States. At the same time, it ensures that individuals have greater control over their health data, with standardized safeguards for data privacy. This regulation seeks to balance the imperative of data solidarity with personal privacy rights. By enabling researchers to quickly obtain multi-country data, the EHDS Regulation is expected to accelerate insights during a health emergency without the delays of negotiating data access case-by-case. The regulation mandates that all EU Member States adhere to common standards, which should improve the quality and reliability of data being shared. While primarily a data governance initiative, the EHDS carries implicit ethical weight as it operationalizes the idea that timely data sharing is essential, as learned in COVID-19, and creates a framework to do this responsibly.

Recently adopted WHO Pandemic Agreement [43], which aims to improve global preparedness and response to future health emergencies. Although it primarily focuses on public health systems and international cooperation, the agreement also emphasized the importance of timely data sharing, equitable access to the benefits of research, including vaccines, diagnostics and treatments and other research ethics issues. By developing shared norms for conducting research during global health crises it signals the recognition of the COVID-19 lessons particularly the need for solidarity, accountability and inclusiveness in health research governance during global crises.

Another important post pandemic document is the WHO guidelines on ethics in health research priority-setting address the recognized need for ethical frameworks in deciding what research gets done, including during health crises [44]. This guidance stresses principles of fairness, transparency, and accountability in research prioritization, asserting that decisions about allocating research funds are value-laden and affect who benefits from future discoveries. It calls for inclusive processes that involve affected communities and various stakeholders in setting research agendas, and for explicit ethical criteria, such as social value and equity impact, to be used alongside scientific merit and feasibility.

Many regulatory agencies have also been updating their guidance to permanently incorporate useful flexibilities from the pandemic [45]. For instance, the FDA and EMA have issued or revised guidelines on decentralized clinical trials, allowing remote consent, telemedicine visits, and mobile labs, which can make trials more resilient during outbreaks.

In light of these findings and developments, some gaps remain to be addressed for future pandemic preparedness. One recurring gap is the need for greater harmonization and clarity among numerous guidelines. While our analysis found a common core, there were also inconsistencies and confusion, especially early in the pandemic, among researchers and RECs about which guidance to follow (international, national or institutional). Moving forward, efforts like the WHO COVID-19 Research Ethics Working Group suggest that having a central, globally endorsed ethical framework at the onset of a crisis can guide local bodies more uniformly. Another challenge is ensuring that ethical guidance keeps pace with scientific innovation. COVID-19 saw the use of novel trial designs (e.g., adaptive platform trials, human infection challenge studies) and ethics guidelines had to adapt to these in real time. Proactively developing ethical guidelines for innovative methodologies, such as the WHO early work on criteria for challenge studies, should be part of preparedness planning.

As mentioned, the COVID-19 pandemic has catalyzed a more integrated view of research ethics, which interweaves participant protection, scientific integrity, and public health needs. One of the initiatives worth mentioning in this context – the PREPARED Code, a Global Code of Conduct for Research during Pandemics [46], a unique ethics guidance framework that integrates research ethics and integrity to break down traditional silos between these disciplines. Unlike standard ethical guidelines, it is specifically tailored to the challenges of pandemic research, acknowledging the heightened ethical dilemmas in such contexts. The concise, jargon-free format, informed by real-world experiences from pandemics, ensures accessibility and practicality. Rooted in values-driven principles, the guidance encourages researchers to act fairly, particularly regarding burdens and inequities during health crises.

## Conclusions

The ethical response to the COVID-19 pandemic has demonstrated both the strengths and limitations of current research ethics frameworks. The COVID-19 pandemic has provided an unprecedented opportunity to re-examine and refine the ethical frameworks that guide research during global health emergencies. The research ethics guidelines developed during the pandemic highlighted the need for flexibility, adaptability, and innovation in the face of a rapidly evolving public health crisis. At the same time, they reinforced the importance of maintaining core ethical principles.

The post-pandemic developments in research ethics also reflect a growing recognition of the need for preparedness, flexibility, and global collaboration in the face of health emergencies. The integration of lessons learned from the COVID-19 pandemic into major ethical guidelines is critical for developing more resilient and adaptable ethical frameworks that can respond to future health emergencies. This includes ensuring that research prioritization mechanisms are in place, that RECs are equipped to handle the challenges of rapid ethical review, and that data-sharing practices are aligned with both scientific openness and the protection of participant privacy.

### Limitations

The study has several limitations that should be considered when interpreting the findings. While the document retrieval strategy was comprehensive, some relevant grey literature or internal institutional guidelines may have been missed, especially those not formally published or not accessible in relevant databases. The inclusion criteria also restricted documents to those available in English, which may have led to the exclusion of relevant research ethics guidelines issued in other languages. Thus, the study reflects the publicly visible layer of ethical governance, not necessarily the full scope of pandemic guidelines, and the language bias could affect the representation of guidelines, limiting the global generalizability of the findings.

## Supplementary Information

Supplementary Material 1.

Supplementary Material 2.

Supplementary Material 3.

## Data Availability

Data is provided within the manuscript or supplementary information files.

## Footnotes

1
https://prepared-project.eu/wp-content/uploads/2024/04/PREPARED-Values-Framework-Final-updated-version.pdf.

2 In this article pandemic ethics documents include guidelines, codes, recommendations and policy briefs adopted by international or national institutions during Covid-19 pandemics.

3 Guidelines adopted in Uganda prioritises research which aims to determine vital aspects that will support timely and effective prevention and control of the pandemic in a sustainable and integrated approach and set a detail list of priorities that should be considered as key areas of research starting form epidemiological surveys, studies on transmission, virological studies, disease models, diagnostic and treatment products, impact of public health measures up to psychological effects, mental health, and other impacts on the society, individuals and patients.
